# De-implementation of healthcare interventions post-COVID-19: evidence from the Evidence-Based Interventions (EBI) programme in England

**DOI:** 10.1136/bmjopen-2024-088256

**Published:** 2026-03-30

**Authors:** Joel Glynn, Tim Jones, Mike Bell, Jane M Blazeby, Christopher Burton, Carmel Conefrey, Jenny Donovan, Nicola Farrar, Josie Morley, Angus GK McNair, Amanda Owen-Smith, Ellen Rule, Gail Thornton, Victoria Tucker, Iestyn Williams, Leila Rooshenas, William Hollingworth

**Affiliations:** 1 Health Economics and Health Policy at Bristol (HEHP), University of Bristol Medical School, Bristol, UK; 2 University of Bristol Musculoskeletal Research Unit, Bristol, UK; 3 NIHR ARC West, Bristol, Bristol, UK; 4 NIHR Bristol Biomedical Research Centre, Bristol, Bristol, UK; 5 School of Allied Health Professions, Canterbury Christ Church University, Canterbury, Kent, UK; 6 University of Bristol Medical School, Bristol, UK; 7 Centre for Surgical Research, Bristol Medical School: Population Health Sciences, University of Bristol Medical School, Bristol, UK; 8 GI Surgery, North Bristol NHS Trust, Bristol, UK; 9 Gloucestershire Integrated Care Board, Gloucester, UK; 10 Public Contributor, Bristol, UK; 11 Bristol, North Somerset and South Gloucestershire ICB, Bristol, UK; 12 University of Birmingham Health Services Management Centre, Birmingham, Birmingham, UK

**Keywords:** HEALTH ECONOMICS, Health policy, SURGERY, Implementation Science, COVID-19

## Abstract

**Abstract:**

**Objectives:**

We explored the effectiveness of the Evidence-Based Interventions (EBI) programme which aimed to reduce the number of inappropriate procedures or diagnostic tests (‘interventions’) in the English National Health Service (NHS). For 12 interventions, we evaluated whether expected intervention counts fell or geographical variation was reduced across England following the publication of EBI Wave 2 guidance in November 2020.

**Design:**

We conducted a retrospective observational study utilising routine hospital data. We applied a controlled interrupted time series analysis to examine both step and trend changes in the use of EBI interventions following the publication of the EBI Wave 2. We compared geographical variation in intervention rates pre-EBI and post-EBI.

**Setting:**

English hospitals where NHS funded elective healthcare interventions are performed.

**Participants:**

Patients who had NHS-funded elective interventions in England from March 2018 to February 2022 inclusive.

**Results:**

Half of the 12 interventions evaluated had significantly lower counts compared with their controls by February 2022. For five of these interventions, counts were between 20% and 57% lower than expected. This was the result of either (1) a larger fall in procedure counts by the end of the pandemic period (n=4 interventions); (2) declining counts post-pandemic (n=1); or (3) a combination of both (n=1). The remaining six interventions had similar or greater than expected counts by February 2022. There was limited evidence of reductions in geographical variation in procedure rates after publication of EBI guidance.

**Conclusions:**

Wave 2 of the EBI programme had a mixed impact on the use of the interventions targeted. There were some successes, however, following significant reduction in the use of elective care during the pandemic, half of the interventions evaluated returned to utilisation at or above predicted levels.

**STRENGTHS AND LIMITATIONS OF THIS STUDY**

- To our knowledge, this is the first evaluation of the Evidence-Based Interventions (EBI) programme’s second wave of guidance for the National Health Service (NHS) in England published in 2020.
- A controlled interrupted time series analysis allowed us to assess the impact of the EBI guidance on surgical procedures while NHS services continued to recover from significant COVID-19 constraints.
- We produced reliable estimates for 12 Wave 2 procedures, using NHS hospital administration data which robustly captures inpatient stays for elective surgery funded by the NHS.
- A further 19 procedures included emergency department, outpatient and diagnostic procedures which are poorly recorded in NHS datasets, were excluded from our analysis.

## Introduction

 Health systems are under pressure to meet the increasing healthcare demands of their populations. For example, in August 2023, 7.7 million people were waiting for elective treatments in the English National Health Service (NHS) compared with 4.4 million in the same month pre-pandemic (2019); more than 395 000 waited for over a year.^1 2^ Therefore, health systems such as the NHS must prioritise treatments and effectively manage waiting lists to maximise the health of their population within the resources available.

A key element of maintaining sustainable health systems is the de-implementation of ongoing healthcare in favour of more cost-effective treatments. De-implementation, also referred to as de-adoption or disinvestment, is the process of stopping or reducing the use of existing healthcare interventions, tests and services.^3^ A number of programmes, including the international ‘Choosing Wisely’ programme and national programmes in both the Netherlands and Australia, have detailed challenges and barriers to the de-implementation of healthcare.^4^,^6^

In 2019, NHS England launched the largest national de-implementation programme in its history. The Evidence-Based Interventions (EBI) programme aimed to reduce the number of ‘inappropriate’ interventions carried out in the NHS.^7^ Inappropriate, in this context, refers to interventions where the benefits do not outweigh the costs, both in terms of patient harm or risk and healthcare resources, in certain circumstances and/or for certain patients. The programme aimed to reduce unwarranted variation, minimise harm and optimise the use of the finite NHS resources. The EBI programme published guidance in four waves. In each wave, guidelines specified clinical criteria for the appropriate use of a number of interventions that are potentially overused in the NHS. Earlier work found the EBI programme to have had little success in reducing intervention rates in the first wave of 17 surgical interventions published in 2019.^8 9^ One reason for this was pre-existing downward trends for many of the chosen interventions left little room for any additional impact from the EBI programme. Furthermore, local NHS commissioners described inconsistent or limited adoption of EBI Wave 1 recommendations. Local policies restricting access to the selected procedures were typically already in place and were either more restrictive or not easily altered to reflect the EBI criteria.^10 11^

The second wave of the EBI programme’s guidance was published in November 2020. This wave included an additional 31 interventions expanding beyond surgical procedures to include both diagnostic tests and outpatient interventions.^12^ To support de-implementation, a web-based system was developed providing access to the criteria, supporting evidence, guidelines and implications, to assist clinicians and patients reach a decision about appropriate care.^12^

Given significant investment in the EBI programme, and the backdrop of record elective care demand in England, it is important to examine its effectiveness. This paper forms part of the OLIVIA study,^13^ a mixed methods evaluation of the EBI programme. The OLIVIA project aimed to understand the delivery, impact and acceptability of the EBI programme, in order to produce recommendations to improve future de-implementation efforts in the NHS and provide key insights for other health systems around the world.

In this paper, we explored whether the EBI programme guidance resulted in the de-implementation and/or reduction in variation of 12 Wave 2 interventions. Specifically, we estimate: (1) any reduction in expected intervention counts by February 2022; (2) whether any reduction in expected intervention counts occurred as a step change and/or as a trend change over time; and (3) whether geographical variation in intervention rates reduced following the introduction of EBI guidelines.

## Methods

### Data sources

Data were extracted from Hospital Episode Statistics Admitted Patient Care (HES-APC) and Outpatient (HES-OP) datasets.^14^ HES-APC is a routinely collected dataset including information from all episodes of NHS-funded hospital-based inpatient and day case care. The dataset includes up to 20 diagnoses (International Classification of Disease (ICD-10)) and records up to 24 procedure codes (Office of Population Censuses and Surveys (OPCS-4)).^15 16^ HES-OP records all specialist consultations in acute hospitals that do not require admission. Increasingly, minor surgical treatments and tests (recorded using OPCS-4 codes) are performed in an outpatient clinic setting. Geography is captured by the Lower Super Output Area (LSOA) of residence for each patient. Using LSOA, we grouped patients into Integrated Care Board areas (ICBs).^17^ There are 42 ICBs in England which are statutory bodies responsible for planning and funding most NHS services for their local populations of up to 4.5 million people.

12 of the 31 interventions selected in Wave 2 of the EBI programme had ICD-10 and OPCS-4 coding data considered sufficiently robust to determine intervention frequencies using routine data.^12^ The remaining 19 interventions are diagnostic tests or outpatient procedures which the EBI programme considered to be unreliably recorded (eg, appendicectomy without confirmation of appendicitis) or not captured (eg, preoperative imaging) in routine healthcare data. Given this, we focused our analysis on the 12 interventions considered robustly measurable (table 1). Full details of all interventions and medical coding are available in EBI guidance documents.^12^

**Table 1:** EBI interventions evaluated

EBI number	Procedure	Description
2A	Diagnostic coronary angiography for low risk, stable chest pain	An invasive X-ray procedure that uses a contrast dye to visualise the coronary arteries.
2B	Repair of minimally symptomatic inguinal hernia	Treatment involves using synthetic mesh to reinforce the abdominal wall.
2C	Surgical intervention for chronic rhinosinusitis	Surgery using a telescope via the nasal cavity to open the sinuses and, if present, remove nasal polyps.
2D	Removal of adenoids for treatment of glue ear	A procedure performed via the mouth to remove excessive adenoidal tissue (adenoidectomy).
2E	Arthroscopic surgery for meniscal tears	Camera and instruments are inserted into the knee through small incisions to repair meniscal tissue.
2G	Surgical removal of kidney stones	Includes a number of techniques such as shockwave lithotripsy, ureteroscopy and percutaneous stone surgery.
2H	Cystoscopy for men with uncomplicated lower urinary tract symptoms	A diagnostic procedure to examine the lining of the bladder and urethra to offer evidence regarding an underlying cause.
2I	Surgical intervention for benign prostatic hyperplasia	Transurethral resection of prostate is a therapeutic procedure involving removal of tissue from the inner aspect of the prostate using diathermy.
2J	Lumbar discectomy	The surgical removal of intervertebral disc material to treat the symptoms resulting from compression of one or more spinal nerve roots.
2K	Lumbar radiofrequency facet joint denervation	Also known as ‘dorsal rhizotomy’ or ‘radiofrequency ablation’, this is a non-surgical and minimally invasive procedure that uses heat to reduce or stop the transmission of pain signals arising from one or more spinal facet joints.
2L	Exercise ECG for screening for coronary heart disease	A type of cardiac stress test.
2M	Upper GI endoscopy	An invasive procedure to examine the lining of the oesophagus, stomach and the first part of the small intestine (duodenum) using a long, flexible tube with a camera.

EBI, Evidence-Based Interventions; GI, gastrointestinal.

Episodes were selected for inclusion in our analyses if the admission or clinic date was between 1 March 2018 and 28 February 2022, allowing at least 2 years of data before the pandemic and publication of the guidelines (in November 2020) to capture pre-intervention rates and trends. We selected episodes of care for each intervention that included the procedure and diagnosis codes, as specified by the EBI programme.^12^ All code lists are available in the supplementary material (online supplemental file S1).

Admitted patient care episodes that included any other elective healthcare intervention were extracted as potential control group interventions. We excluded: episodes with interventions identified in earlier EBI guidelines (Wave 1) and their potential substitutes^9^; EBI Wave 2 interventions; and cancer-related treatments (cancer exclusion codes available in online supplemental file S1).

### Missing data

The recording of procedure codes in HES-APC administrative data is considered very high quality and is linked to the payment/reimbursement of hospital trusts in the English NHS.^14^ However, as with any routine dataset, miscoding does occur. Age and sex, used in the standardisation of process, are well recorded in HES (<0.5% and <0.1% missing) so no imputation was necessary.

### Analysis period

**Figure 1:**
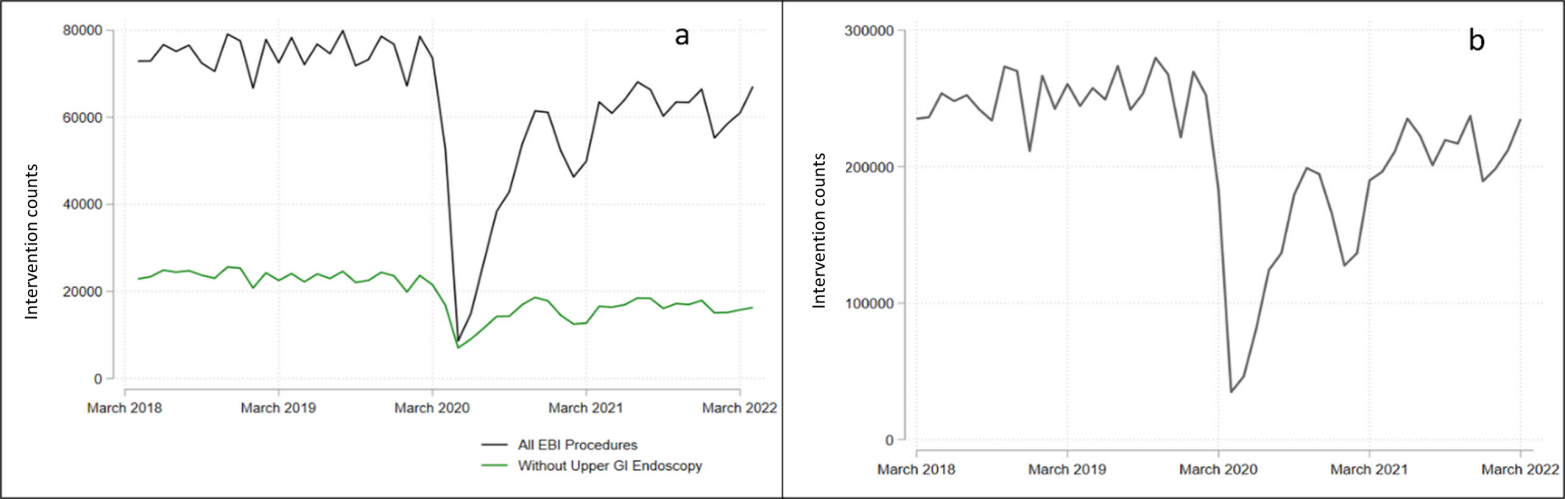
Pooled monthly intervention counts for EBI (a) and non-EBI elective procedures (b). GI, gastrointestinal.

We conducted analyses across three periods, pre-EBI-COVID (1 March 2018 to 29 February 2020), COVID-19 (1 March 2020 to 28 February 2021) and post-EBI-COVID (1 March 2021 to 28 February 2022). The start of the COVID-19 period was selected as March 2020, the first month with COVID-19 lockdown restrictions in England. We considered the end of the intensive COVID-19 period (28 Feb 2021) to be the point after which restrictions had mostly lifted and elective intervention counts levelled out following the second wave of the pandemic (figure 1b).

**Figure 2:**
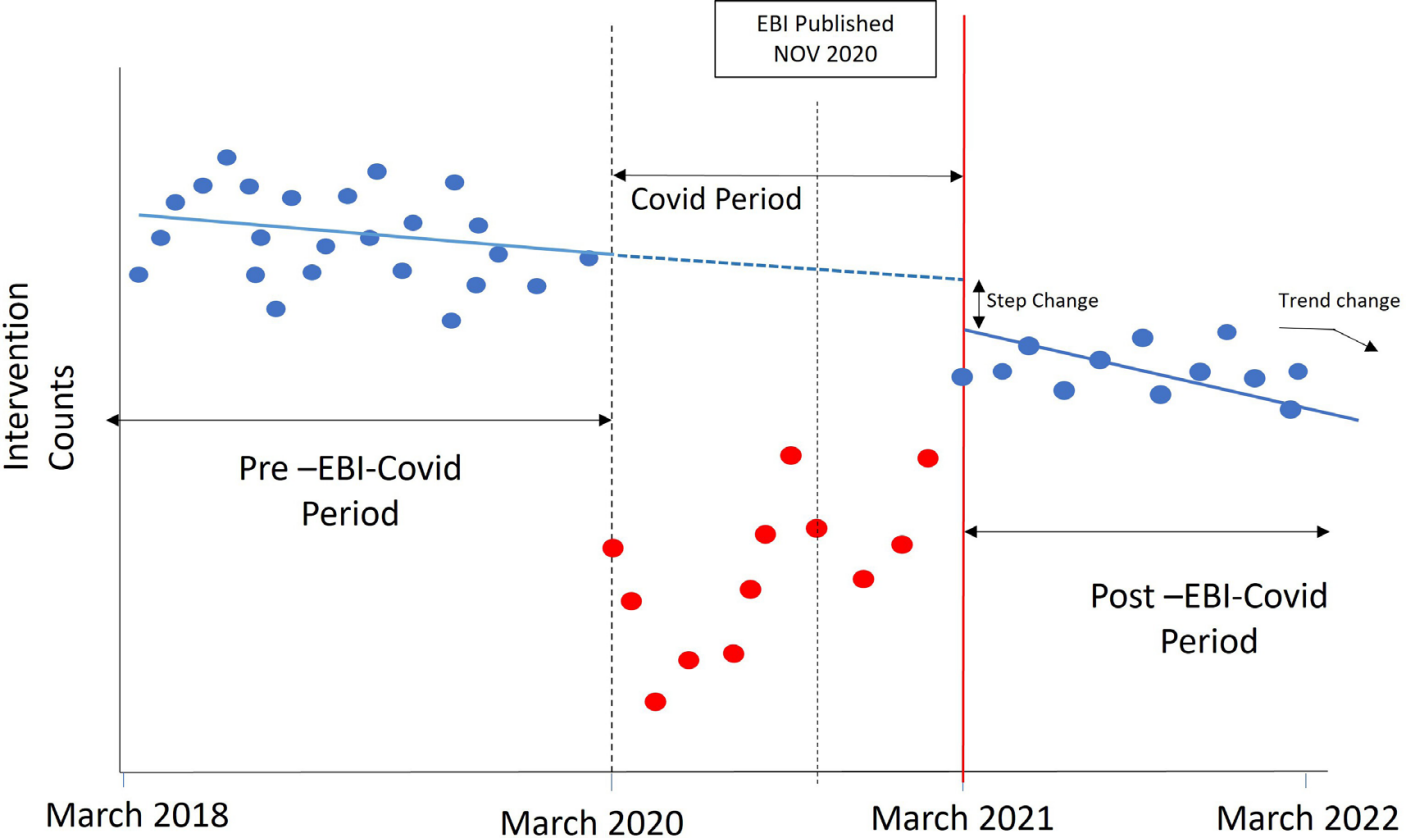
Illustrative depiction of analyses of the impact of EBI on intervention counts. EBI, Evidence-Based Interventions.

Beginning in March 2020, there were three separate national ‘lockdowns’ in England aiming to reduce the spread of COVID-19, with gradual release from lockdown in early 2021; these resulted in vast reductions in elective hospital care (figure 1b). To ‘factor out’ the impact of COVID-19 on elective interventions in the NHS, we excluded all data points during the intensive COVID-19 period (indicated in red in figure 2), assuming a continued pre-EBI-COVID trend during this period (blue dashed line in figure 2).

### Control group

We generated unique control groups for each EBI Wave 2 intervention using the pool of elective interventions not considered for de-implementation, excluding cancer admissions. We used the Stata ‘*synth*’ command to generate control groups by selecting weighted averages of these interventions (captured as three-character OPCS codes).^18^ This method created synthetic control groups that best resembled each EBI intervention in the pre-EBI-COVID period based primarily on the trend, but also taking account of monthly intervention counts, the proportion of women treated and average patient age.^19^

### Statistical analyses

All analyses were carried out in Stata V.17 statistical software.^20^

#### Controlled interrupted time series analysis

To explore whether trends in monthly intervention counts changed following the publication of the EBI guidance, we performed a controlled interrupted time series (CITS) analysis.^21^ This analysis estimated whether there was a step (immediate) or trend (over time) change for each EBI intervention during the post-EBI-COVID period (ie, 1 March 2021 onwards), compared with their control group.

Monthly counts for each control group were subtracted from their matched EBI intervention counts. An interrupted time series analysis was run using segmented linear regression on these differenced counts, with 1 March 2021 as the interruption point, to estimate step-change and trend-change coefficients for the CITS analyses. We included a winter month dummy variable to account for the usual seasonal fluctuations in elective interventions.

Using the control intervention trend from the model as a prediction of what would have happened without the EBI guidance, we generated a ‘1-year prediction’ that estimated the impact of the EBI programme 1 year after the COVID-19 period (ie, EBI intervention count minus control group count in February 2022). Negative ‘1-year predictions’ provide evidence that the EBI programme was successful in achieving de-implementation, taking into account the impact of the pandemic which affected all elective interventions.

#### Variation analyses

We compared intervention rates by ICB pre-EBI-COVID and post-EBI-COVID. Indirectly age-sex standardised intervention rates per 100 000 residents were calculated for each year, taking account of population, age and sex differences between ICBs.^22^ Expected intervention counts for each ICB were estimated using the national age/sex-specific intervention rates (per population) applied to the age/sex-specific ICB populations. Two metrics were used to compare variation: the 90/10 ratio and the systematic component of variation (SCV).^23^ The 90/10 ratio quantifies the difference between the intervention rates of the ICB at the 90th and the 10th percentiles; a larger value represents greater variation. The SCV statistic takes account of chance variation and incorporates the more extreme values excluded by the 90/10 ratio.^24^ We generated 95% CIs around SCV statistics using the percentile method bootstrapping with 1000 replications.

### Sensitivity analyses

#### Analysis periods changes

There are no universally agreed dates for when the effect of COVID-19 on elective interventions began or ended in England; therefore, we re-ran the CITS model with (1) an earlier start date (1 February 2020) for the COVID-19 period; (2) a prolonged COVID-19 period (1 March 2020 to 31 May 2021); and (3) an earlier interruption point (1 November 2020) assessing if the EBI guidance had immediate impact.

#### Alternative control groups

We derived alternative control groups based on a clinical guide to surgical priorities during the COVID-19 pandemic which identified the ‘lowest priority’ interventions during the pandemic.^25^ For EBI interventions in specialties with a high volume of ‘lowest priority’ interventions (urology; musculoskeletal; ear, nose and throat), we pooled all ‘lowest priority’ interventions in the relevant specialty as the control group. EBI interventions in other specialties were compared with a control group comprised of all ‘lowest priority’ interventions regardless of specialty (see online supplemental file 2). We scaled the control group intervention rates to a similar level of monthly activity of each EBI intervention, using a multiplier generated by differencing average counts in the pre-EBI-COVID period for both the EBI and control counts.

#### Patient and public involvement

GT and MB are named authors and public contributors to this work. Both have been involved in the work since its inception, including the development of the research project, interpretation of results and editing of the final manuscript. The study plan and subsequent results have been presented to the patient and public advisory group of our wider mixed-method project (NIHR130547), gaining insight into the interpretation of results.^13^

## Results

### Overview of intervention counts

**Figure 3:**
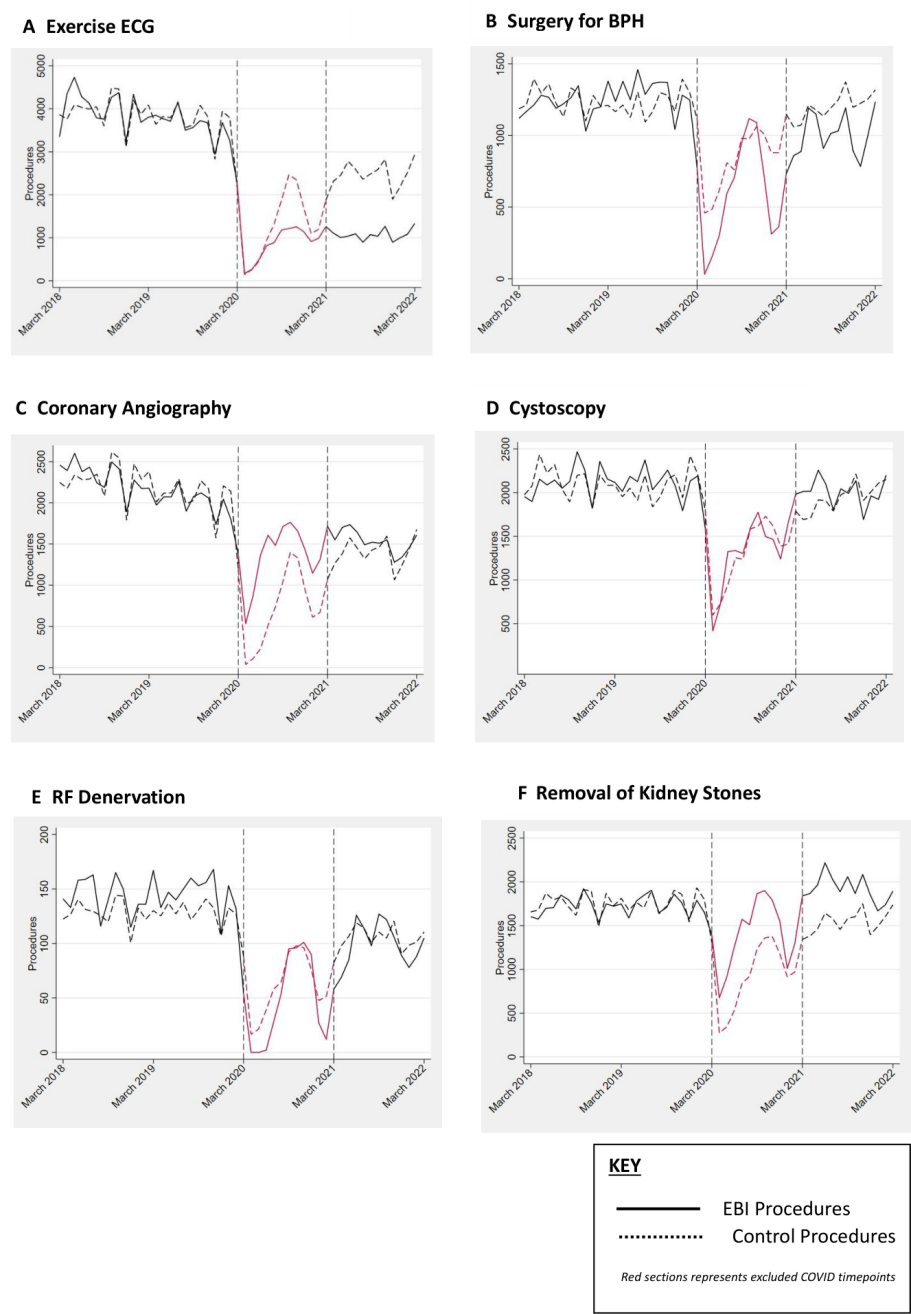
Time series plots of 6 of 12 EBI interventions and controls. BPH, benign prostatic hyperplasia; EBI, Evidence-Based Interventions, RF, radiofrequency.

Pre-EBI and COVID-19, there were a total of 891 031 and 901 671 EBI interventions performed in 2018/2019 and 2019/2020, respectively (figure 1a, black line). This fell to 509 571 during the intensive COVID-19 period (2020/2021), increasing back towards pre-COVID-19 levels afterwards (751 482 interventions in 2021/2022). Upper gastrointestinal (GI) endoscopy accounted for the majority (74%) of the EBI interventions measured. However, a similar trend, a large fall during COVID-19 followed by a partial recovery in intervention counts was observed for other EBI interventions (figure 1a, green line) and all other non-EBI elective procedures (figure 1b). Pre-EBI and COVID-19, the control group’s monthly counts were well matched to the EBI procedure counts except for upper GI endoscopy in which the intervention counts were much higher than the control (figure 3 and online supplemental figure 1).

### Changes in intervention counts post-EBI-COVID

6 of 12 interventions (exercise ECG, surgery for chronic rhinosinusitis, lumbar discectomy, adenoids removal surgery for benign prostatic hyperplasia (BPH) and arthroscopic surgery for arthroscopic surgery for meniscal tears; table 2, first column in green) selected by the EBI programme had significantly lower intervention counts in February 2022 than would have been expected based on trends in the matched control groups. The largest absolute and percentage differences in expected intervention counts were seen in exercise ECG with 1573 (95% CI 1742 to 1404) (57%) fewer interventions than the matched control. In a further four interventions, reductions ranged from 20% to 45%. The difference in arthroscopic surgery for meniscal tears, although statistically significant, represented less than a 1% reduction in expected procedure counts.

**Table 2:** CITS regression results ranked by the difference in 1-year prediction

EBI procedure	1-year prediction (95% CI)	% difference Feb 2022*	Step change (95% CI)	Trend change 95% (CI)
Exercise ECG for screening for CHD	−1573† (−1742 to −1404)	−57	−613‡ (−1200 to −26)	−10 (−59 to 39)
Surgery for chronic rhinosinusitis	−252‡ (−298 to −207)	−36	−249‡ (−398 to −101)	−7.4 (−20 to 6)
Surgery for BPH	−103‡ (−171 to −34)	−20	−543‡ (−750 to −335)	0.3 (−16 to 17)
Diagnostic coronary angiography	−65 (−161 to 31)	−4	798‡ (553.3 to 1042.7)	−27‡ (−46 to −8)
Cystoscopy	−50 (−191 to 92)	−9	124 (−222 to 469)	−37‡ (−59 to −15)
Lumbar discectomy	−43‡ (−57 to −29)	−41	−20 (−60 to 19)	−4‡ (−7 to −2)
Adenoids removal	−34‡ (−40 to −28)	−45	−24† (−47 to 0)	4‡ (2 to 5)
Arthroscopic surgery for meniscal tears	−21‡ (−42 to 0)	<1	−21 (−67 to 25)	−2 (−5 to 1)
RF denervation	4 (−9 to 18)	−13	−43‡ (−65 to −22)	1 (−1 to 3)
Hernia repair	63 (−180 to 306)	−9	−43‡ (−65 to −22)	10 (−56 to 77)
Removal kidney stones	231‡ (165 to 297)	+8	623‡ (464 to 783)	−31‡ (−43 to −19)
Upper GI endoscopy	3665‡ (1433 to 5896)	+12	2003 (−2017 to 6023)	−493‡ (−875 to −111)

Green represents statistically significant evidence of de-implementation; grey no statistically significant evidence of adoption; and red statistically significant evidence of greater adoption than would be expected compared with the control group.

† p-value <0.05

‡ p-vlaue <0.01

* Percentage difference between the EBI procedure and its control in February 2022.

BPH, benign prostatic hyperplasia; CHD, coronary heart disease; EBI, Evidence-Based Interventions; GI, gastrointestinal; RF, radiofrequency.

In four of these six interventions, exercise ECG, surgery for BPH (table 2 and figure 3), surgery for chronic rhinosinusitis and adenoid removal (table 2 and online supplemental figure 2), a larger fall in intervention counts (ie, step change) compared with the control group was evident at the end of the pandemic period. In one intervention, lumbar discectomy (online supplemental file 1, figure 3), intervention counts compared with the control declined post-pandemic (ie, trend change). In the final intervention, arthroscopic surgery for meniscal tears (online supplemental file 1, figure 3), the difference was due to a combination of small step and trend changes.

In contrast, for two interventions (removal of kidney stones and upper GI endoscopy) counts in February 2022 were higher than predicted (table 2; first column in red). For both, this was due to a relatively smaller fall (step-change) in interventions than predicted during the COVID-19 period. However, for both interventions, there was a relative downward trend in intervention rates post-pandemic. The remaining four interventions (coronary angiography, cystoscopy, radiofrequency (RF) denervation and hernia repair) had non-significantly different 1-year predictions (table 2; first column in grey).

### Geographical variation

The majority of interventions had relatively low geographical variation pre-EBI-COVID with no substantial reduction observed afterwards (table 3). The largest pre-EBI-COVID variation was found in RF denervation (90/10 ratio of 15.7; table 3), cystoscopy (9.6) and exercise ECG (7.1). For RF denervation, there was a reduction in variation post-EBI-COVID (90/10 ratio difference of −4.99; table 3). The SCV score for RF denervation also decreased post-EBI-COVID, although CIs were wide and included zero (−8.1; 95% CI −63.9 to 36.4; online supplemental table S1).

**Table 3:** Intervention rate variation across English ICBs: 90/10 ratios by year

EBI procedure	Pre-EBI-COVID 2019/2020	COVID-19 2020/2021	Post-EBI-COVID 2021/2022	Difference 2019/2020– 2021/2022
Exercise ECG for screening for CHD	7.10	8.28	7.14	0.04
Surgery for chronic rhinosinusitis	2.21	2.98	2.84	0.63
Surgery for BPH	1.42	1.71	1.57	0.15
Diagnostic coronary angiography	1.78	1.92	1.80	0.02
Cystoscopy	9.66	8.56	8.84	−0.18
Lumbar discectomy	3.31	6.41	5.11	1.80
Adenoids removal	4.07	3.90	6.14	0.15
Arthroscopic surgery for meniscal tears	2.33	3.00	3.05	0.71
RF denervation	15.66	11.54	10.67	−4.99
Hernia repair	1.31	1.95	1.80	0.02
Removal of kidney stones	1.74	1.66	1.55	−0.18
Upper GI endoscopy	1.51	1.64	1.61	0.10

BPH, benign prostatic hyperplasia; CHD, coronary heart disease; EBI, Evidence-Based Interventions; GI, gastrointestinal; ICBs, Integrated Care Boards ; RF, radiofrequency.

### Sensitivity analyses

#### Analysis periods changes

The sensitivity analyses altering the COVID-19 period dates, or the EBI interruption point supported our primary findings (online supplemental table S2).

#### Alternative control group

Analyses using the ‘lowest priority’ intervention control groups, supported evidence of lower intervention counts than expected in February 2022 for five of the six interventions identified in the primary analysis (exercise ECG, chronic rhinosinusitis, lumbar discectomy, adenoids removal and meniscal tears; online supplemental table S3, figure S2). However, surgery for BPH had evidence of higher intervention counts than predicted compared with the urology ‘lowest priority’ control group.

## Discussion

As was evident across all elective healthcare in England, the use of 12 interventions targeted by the EBI programmes Wave 2 guidelines had not returned to pre-COVID-19 levels by February 2022. After controlling for the impact of the pandemic on all elective care, 6 of 12 interventions had lower than expected intervention counts by February 2022. In four of these procedures, this fall was greater than 35%. The remaining six interventions had counts at a similar (n=4) or higher (n=2) level than predicted.

In four of the six interventions, the lower than expected counts were predominantly due to a larger fall in procedure counts by the end of the pandemic period; for one, it was declining counts post-pandemic restrictions, and in the final one, a combination of both. Consequently, there is some evidence to support the hypothesis that the EBI programme led to de-implementation in some of the interventions targeted.

For many interventions, there was relatively low geographical variation in intervention rates pre-EBI and little or no reduction in variation afterward. Therefore, there was very limited evidence that the publication of EBI guidelines minimised variation in care.

### Strengths and limitations

#### Strengths

To our knowledge, this is the first analysis of the EBI programme’s second wave of guidance. This work provides insights into a national de-implementation initiative at a time when services were under pressure and when the opportunity cost of undertaking ‘inappropriate’ interventions was very high. HES data makes it possible to precisely count all elective admissions for interventions funded by the NHS in England.^14^ We were therefore able to accurately evaluate nationwide intervention counts and variation over time. Although some patient characteristics are not reported consistently in the HES dataset (eg, ethnicity), the variables used for this analysis, including diagnosis and procedure codes, are very well-recorded in the inpatient setting.^14^ The CITS analysis allowed comparison of intervention counts between interventions targeted by the EBI programme and those which were not in order to identify the effects of the de-implementation initiative.

#### Limitations

Although our methods aimed to minimise the confounding effect of COVID-19, we cannot eliminate it. The pandemic-related reductions in all elective surgery may have provided an opportunity for health systems to re-assess and de-implement procedures already viewed as of lower value. However, our findings were largely consistent in sensitivity analyses when ‘lowest priority’ interventions not targeted by the EBI programme were used as control groups. This suggests that, at least for some procedures, EBI guidance may have played an additional role in reducing re-uptake post-pandemic. Other concurrent factors, such as the publication of trial results or clinical guidelines, may also have affected the use of specific procedures.

There are no definitive start and end points for COVID-19 and its impact on health services. We used time points based on visual inspection of elective intervention counts over time. However, in sensitivity analyses varying these time points, we found little change in our results. Given the lack of reliable prevalence data for the health conditions treated by the interventions, we evaluated intervention counts rather than rates. The underlying prevalence of health conditions may have fluctuated over the 4-year analysis period, which could have impacted procedure counts. It is well documented that the COVID-19 pandemic increased the global burden of many non-COVID-19 conditions such as mental health and heart disease.^26^ However, we have no reason to believe that the health conditions associated with EBI interventions would have a differential change in prevalence compared with health conditions associated with the control interventions.

The synthetic control method allowed us to create control groups that closely matched EBI intervention trends pre-EBI-COVID for almost all interventions. However, the very high monthly counts for upper GI endoscopy limited the ability of this approach to select an appropriate control group for this procedure. Our findings may be less reliable for this intervention, although we observed similar findings in sensitivity analysis using a ‘lowest priority’ control group for this intervention.

We were only able to evaluate 12 of the 31 procedures targeted in Wave 2, providing a detailed but potentially limited picture of the overall effectiveness of the EBI programme. Routine NHS data lacked the reliability or coding detail necessary to track a further 13 procedures. For example, the capture of Troponin testing in the Emergency Care Data Set in England is relatively new; therefore, increases in usage may relate to improvements in data collection over time rather than true increases in its use. The remaining six interventions are not currently captured in any routine healthcare dataset. However, alongside our evaluation of Wave 1,^9^ analyses exploring healthcare managers views and local policy changes in response to the EBI programme,^10 11^ and further work to come, we provide a broad picture of the successes and challenges of a complex national de-implementation programme.

### Comparisons with other literature

The de-implementation literature to date has focused on evaluation of interventions to de-implement individual medical procedures or tests. Cliff *et al* systematically reviewed multiple single interventions within the Choosing Wisely programme in the USA between 2012 and 2019.^4^ They found that interventions with multiple active de-implementation components (eg, clinician education and shared decision-making) were much more likely to be effective than those that only disseminated Choosing Wisely recommendations.

A process evaluation of the Dutch ‘to do or not to do?’ programme (2016–2018) showed that reductions in ‘inappropriate’ care are possible.^6^ The programme selected eight ‘de-implementation projects’ with bespoke de-implementation strategies, such as education of general practitioners, appointing clinical champions and changes to pathways of care. The evaluation concluded that five out of eight projects resulted in a reduction of inappropriate care and identified barriers (eg, lack of time) and facilitators (eg, support among clinicians) for success.

Further qualitative work, following on from recently published work exploring the views of local commissioners,^10^ is soon to be published, unpicking the reasons for the impact or apparent lack of impact of the EBI programme from the perspective of patients and clinicians as part of our wider project (NIHR130547).^13^

### Implications

Given the ongoing constraints on healthcare expenditures and the need to provide financial ‘headroom’ for effective but costly new healthcare, it is essential that de-implementation initiatives are designed and executed well. The COVID-19 pandemic, which at its peak resulted in a 95% reduction in elective hospital care, provided a unique opportunity to reconsider the appropriate use of healthcare resources. However, as we have demonstrated, even in these circumstances, the dissemination of guidelines on the appropriate use of interventions did not have a universal impact on de-implementation or reduce geographical variation in the uptake of care. Our current findings on EBI Wave 2 are more encouraging than previous work on Wave 1^8 9^ which did not identify any impact. This may be due to Wave 2 focusing on interventions not already decreasing pre-guidance. Nevertheless, our results are mixed and many questions remain about the mechanisms by which de-implementation programmes work and why they work in some circumstances and not others. In this work, we see the largest reductions in use of exercise ECG, which was the only procedure with a ‘do not do’ recommendation. This may have resulted in a simpler de-implementation message and process in comparison to implementing nuanced criteria for appropriate use. As de-implementation programmes require significant resources, it is very important that they are robustly evaluated, even when outcomes are difficult to measure using routine data. The value of future de-implementation initiatives depends on learning from the successes and failures of current programmes.

### Future research

This work is part of a larger body of research examining the de-implementation of inappropriate care using the NHS EBI programme as a case study (NIHR130547).^9^,^1113^ We are also researching the spillover effects of de-implementation in the primary care setting, including the impact on referrals to secondary care and non-surgical treatment. Our team is also undertaking qualitative work investigating patients’, clinicians’ and commissioners’ accounts of the actions taken in response to the EBI programme, and the perceived consequence of this on the delivery and experience of care. The findings will provide insight into potential mechanisms by which the EBI programme reduced activity rates, reasons why it did not (where applicable), and the wider consequences for delivery and receipt of care.

### Conclusions

We found evidence of a reduction in 6 of 12 of the Wave 2 EBI interventions analysed. De-implementation of healthcare remains challenging. Even following a period of forced reductions in elective interventions resulting from the pandemic, half of the interventions analysed returned to levels at or above what would have been expected without the EBI programme. There was little evidence that the publication of national criteria for the appropriate use of surgical interventions reduced variation in procedures across England.

## Supplementary material

10.1136/bmjopen-2024-088256 online supplemental file 1

10.1136/bmjopen-2024-088256 online supplemental file 2

## Data Availability

Data may be obtained from a third party and are not publicly available.

## References

[1] NHS England and Improvement (2019). Referral to Treatment (RRT) Waiting Times - Monthly RRT waiting times for incomplete pathways.

[2] NHS England (2023).

[3] Niven DJ, Mrklas KJ, Holodinsky JK (2015). Towards understanding the de-adoption of low-value clinical practices: a scoping review. BMC Med.

[4] Cliff BQ, Avanceña ALV, Hirth RA (2021). The Impact of Choosing Wisely Interventions on Low-Value Medical Services: A Systematic Review. Milbank Q.

[5] Harris C, Allen K, Ramsey W (2018). Sustainability in Health care by Allocating Resources Effectively (SHARE) 11: reporting outcomes of an evidence-driven approach to disinvestment in a local healthcare setting. BMC Health Serv Res.

[6] Verkerk EW, van Dulmen SA, Westert GP (2022). Reducing low-value care: what can we learn from eight de-implementation studies in the Netherlands?. BMJ Open Qual.

[7] (2024). Academy of Medical Royal Colleges.

[8] Anderson M, Molloy A, Maynou L (2023). Evaluation of the NHS England evidence-based interventions programme: a difference-in-difference analysis. BMJ Qual Saf.

[9] Glynn J, Jones T, Bell M (2023). Did the evidence-based intervention (EBI) programme reduce inappropriate procedures, lessen unwarranted variation or lead to spill-over effects in the National Health Service?. PLoS One.

[10] Farrar N, Conefrey C, Bell M (2025). Relevance and flexibility are key: exploring healthcare managers’ views and experiences of a de-adoption programme in the English National Health Service. BMC Health Serv Res.

[11] Conefrey C, Farrar N, Coyle M (2025). Delivering a national de-adoption programme: a documentary analysis of local commissioning policy compliance with England’s Evidence-based Interventions programme (EBI). BMC Health Serv Res.

[12] Academy of Medical Royal Colleges (2020). Evidence-based interventions list 2 guidance.

[13] NIHR (2023). NIHR Funding and Awards: NIHR 130547.

[14] Herbert A, Wijlaars L, Zylbersztejn A (2017). Data Resource Profile: Hospital Episode Statistics Admitted Patient Care (HES APC). Int J Epidemiol.

[15] World Health Organization (2004). International Statistical Classification of Diseases and Related Health Problems: Tenth Revision.

[16] NHS Digital (2021). National Clinical Coding Standards OPCS-4.

[17] Office for National Statistics (2022). LSOA (2011) to Sub ICB Locations to Integrated Care Boards to Local Authority Districts (july 2022) Lookup in England.

[18] Abadie A, Diamond A, Hainmueller J (2011). Statistical Software Componants S457334.

[19] Linden A (2018). Combining synthetic controls and interrupted time series analysis to improve causal inference in program evaluation. J Eval Clin Pract.

[20] StataCorp LLC CS TX.stata statistical software: release 17.

[21] Lopez Bernal J, Cummins S, Gasparrini A (2018). The use of controls in interrupted time series studies of public health interventions. Int J Epidemiol.

[22] Analysis Group of PAHO’s Special Program for Health Analysis (SHA) (2002). Standardization: A Classic Epidemiological Method for the Comparison of Rates. Epidemiol Bull.

[23] Ibáñez B, Librero J, Bernal-Delgado E (2009). Is there much variation in variation? Revisiting statistics of small area variation in health services research. BMC Health Serv Res.

[24] Friebel R, Hauck K, Aylin P (2018). National trends in emergency readmission rates: a longitudinal analysis of administrative data for England between 2006 and 2016. BMJ Open.

[25] Federation of Surgical Speciality Association (FSSA) (2020). Clinical guide to surgical prioritisation during the coronavirus pandemic.

[26] Chen C, Zhou W, Cui Y (2025). Global, regional, and national characteristics of the main causes of increased disease burden due to the covid-19 pandemic: time-series modelling analysis of global burden of disease study 2021. BMJ.

