# Supplementary material for: De-implementation of healthcare interventions post-COVID-19: evidence from the Evidence-Based Interventions (EBI) programme in England
Source: BMJ Open. 2026 Mar 30;16(3):e088256. doi: 10.1136/bmjopen-2024-088256 (PMC13052614; doi:10.1136/bmjopen-2024-088256)
Supplement: online supplemental file 2 [file bmjopen-16-3-s002.docx]

# De-adoption of healthcare interventions post-COVID: Evidence from the Evidence-Based Interventions (EBI) programme in England.

Joel Glynn^1*^, Tim Jones^1,2^, Mike Bell^3,4^, Jane Blazeby^4^, Christopher Burton^6^, Carmel Conefrey^5^, Jenny L. Donovan^5^, Nicola Farrar^5^, Josie Morley^1^, Angus McNair^5,7^, Amanda Owen-Smith^1^, Ellen Rule^8^, Gail Thornton^5^, Victoria Tucker^9^, Iestyn Williams^10^, Leila Rooshenas^5^ and William Hollingworth^1^.

# Online Supplementary Material S2


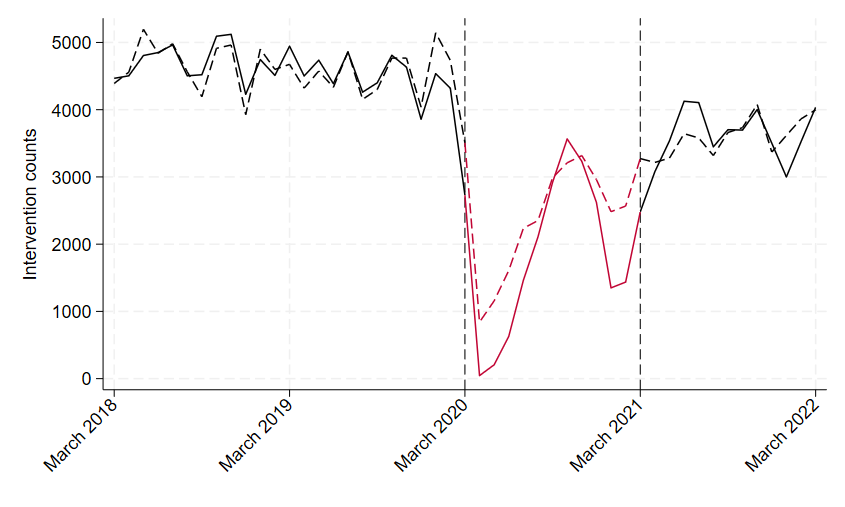


**J. Meniscal Tears**

**H Chronic Rhinosinusitis**


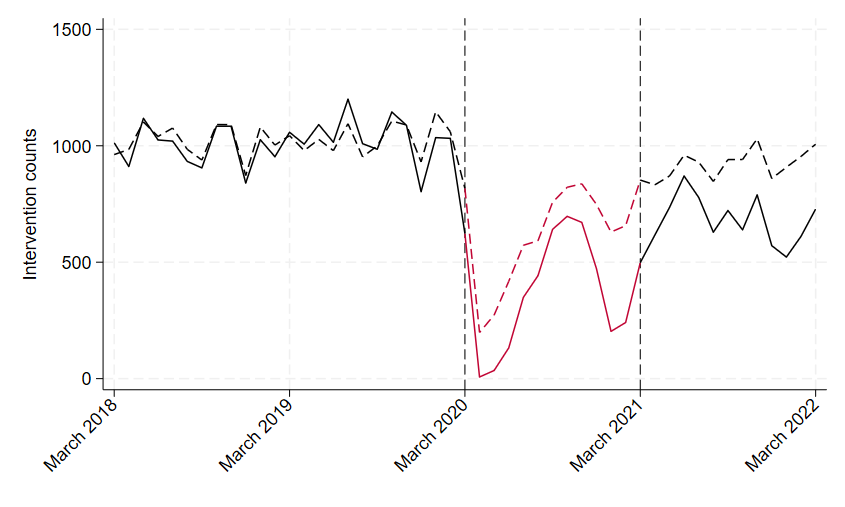

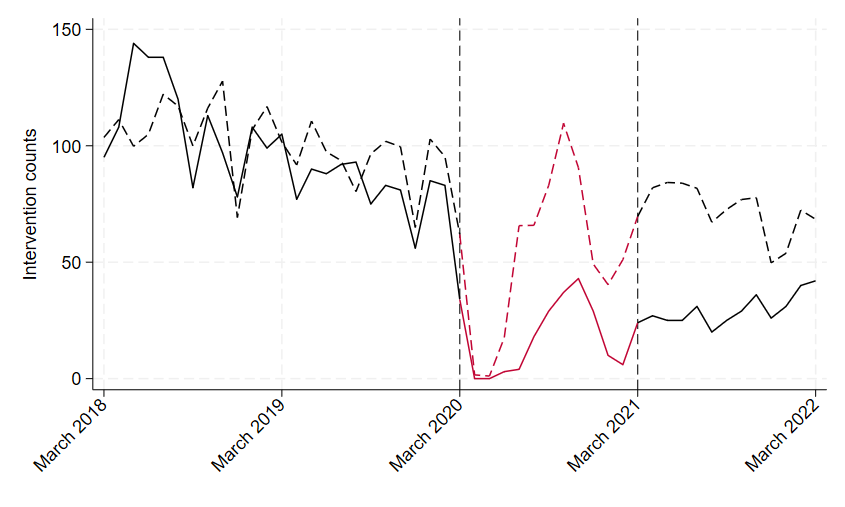

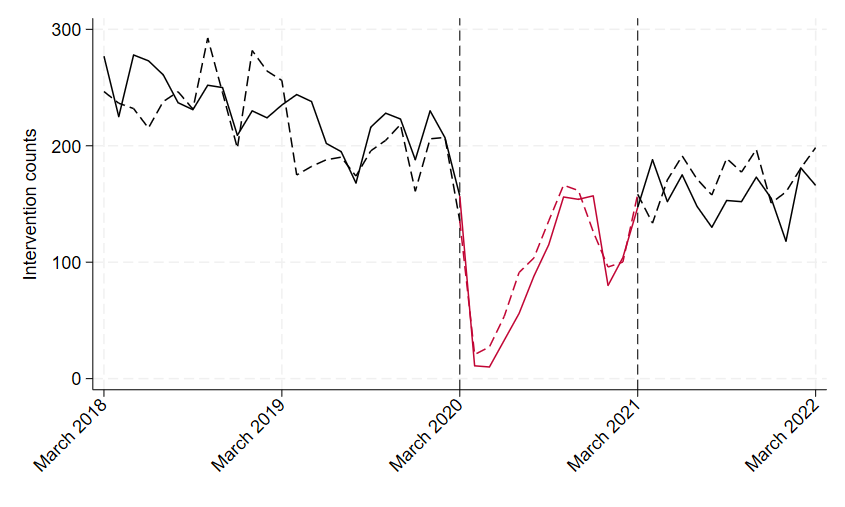

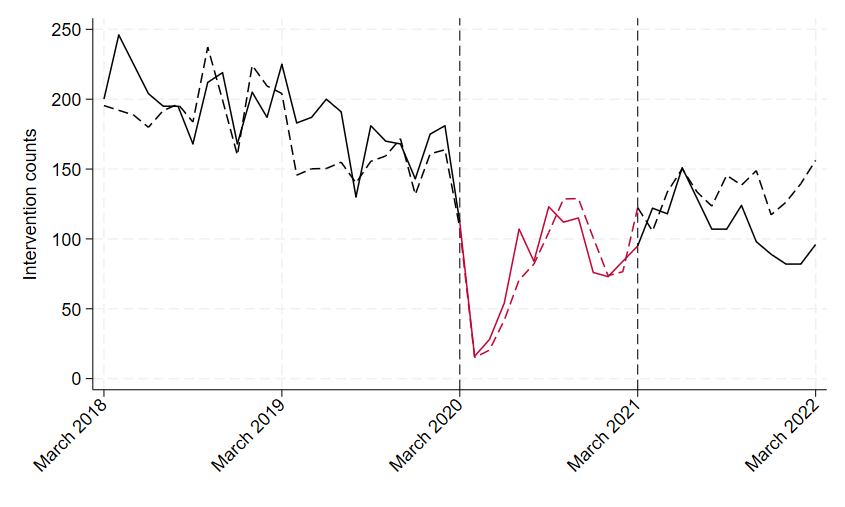

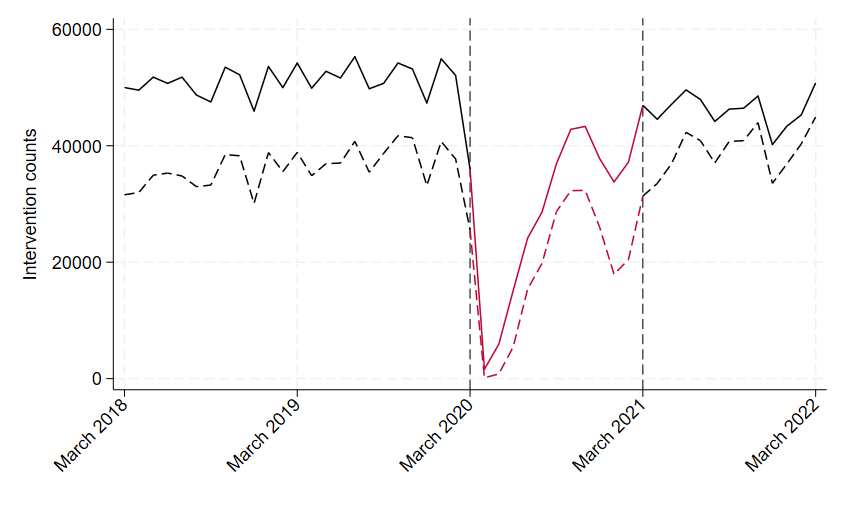


**I. Adenoids Removal**

**G. Hernia Repair**

**K. Lumbar Discectomy**

**G. Upper GI Endoscopy**

Figure S1 Timeseries plots of EBI intervention and related controls remaining 6 of 12

**KEY**

EBI Procedures

Control Procedures

*Red sections represents excluded COVID timepoints*

Figure S2 Time series plots of all 12 EBI intervention and **Scaled** Alternative Controls (Priority 4 Procedures)

1.
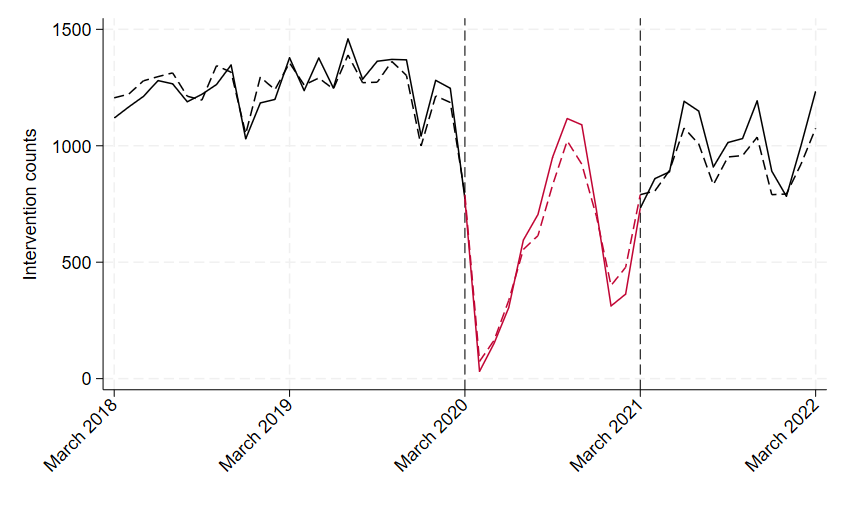

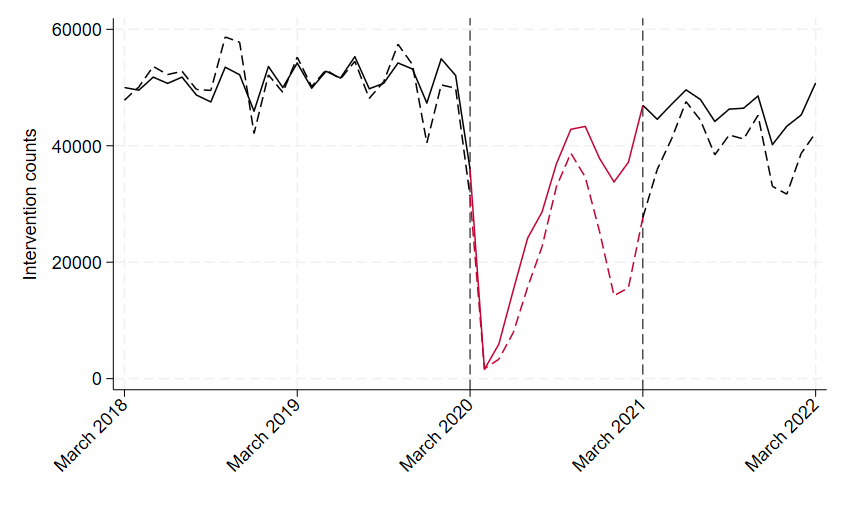
**Exercise ECG (All Cat.4 Control Scaled) B. Surgery for BPH (Urology Car.4 Control)**
2. **Coronary Angiography (All Cat.4 Control) D. Cystoscopy (Urology Cat.4 Control)**

**
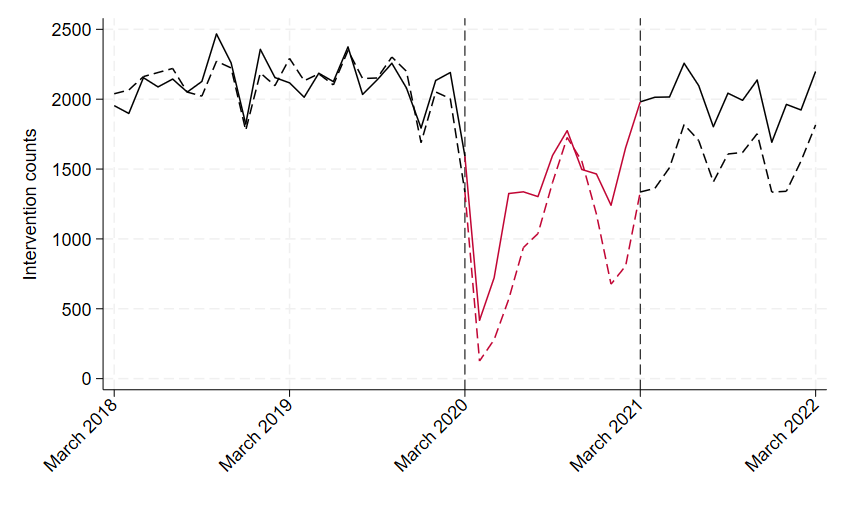

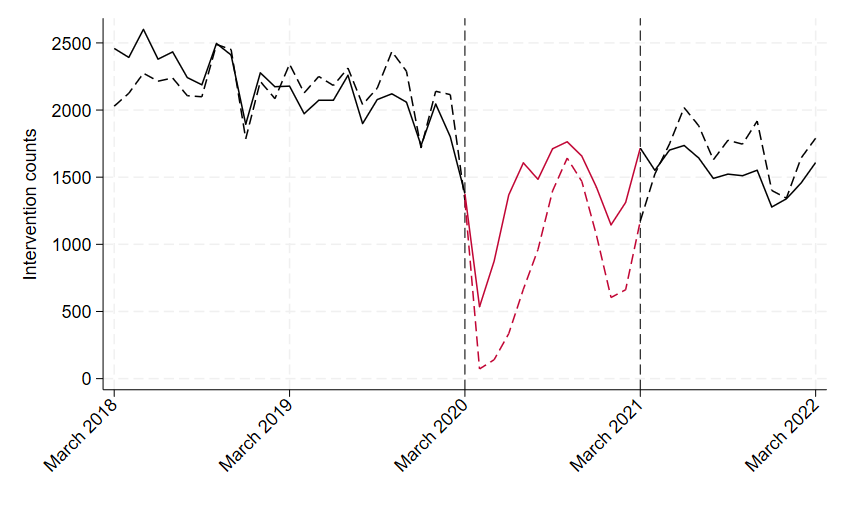
**


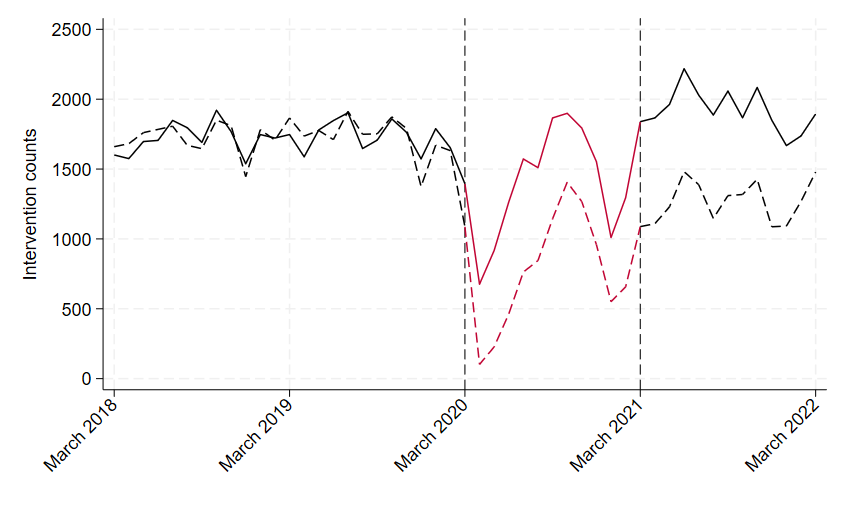
**
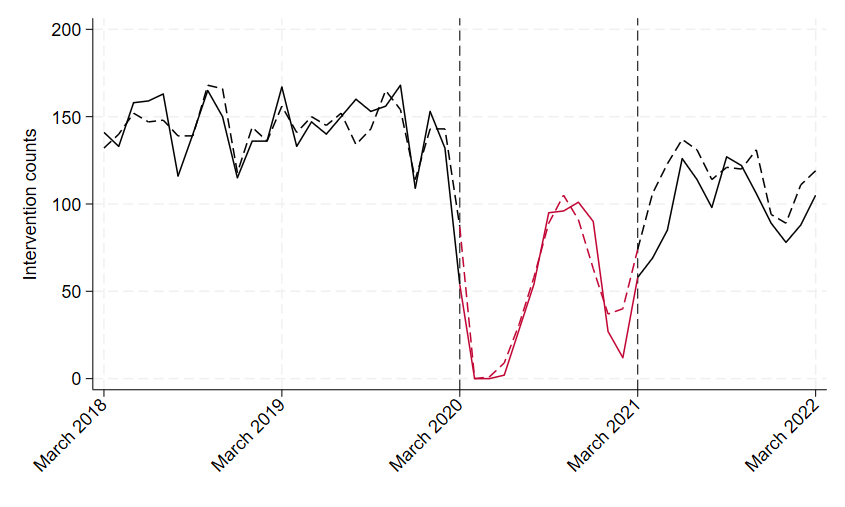
 E. RF Denervation (MSK Cat. 4 Control) F. Removal of Kidney Stones (Urology Cat.4 Control)**

**
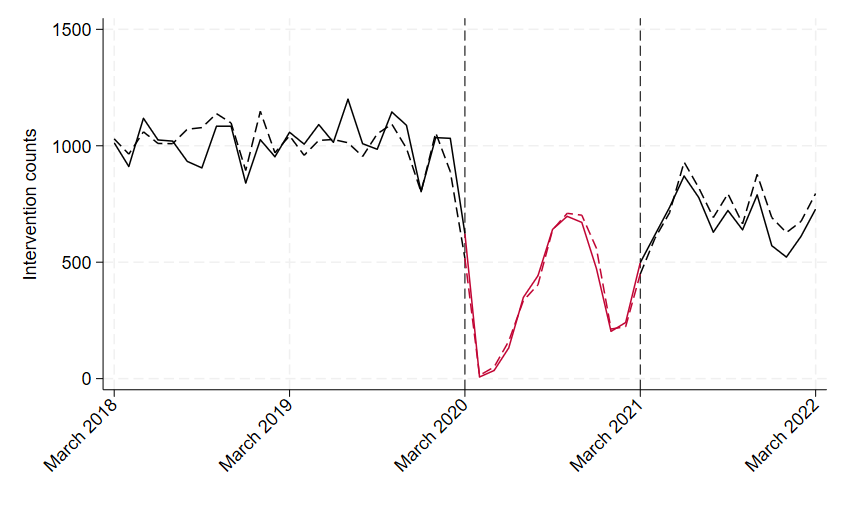

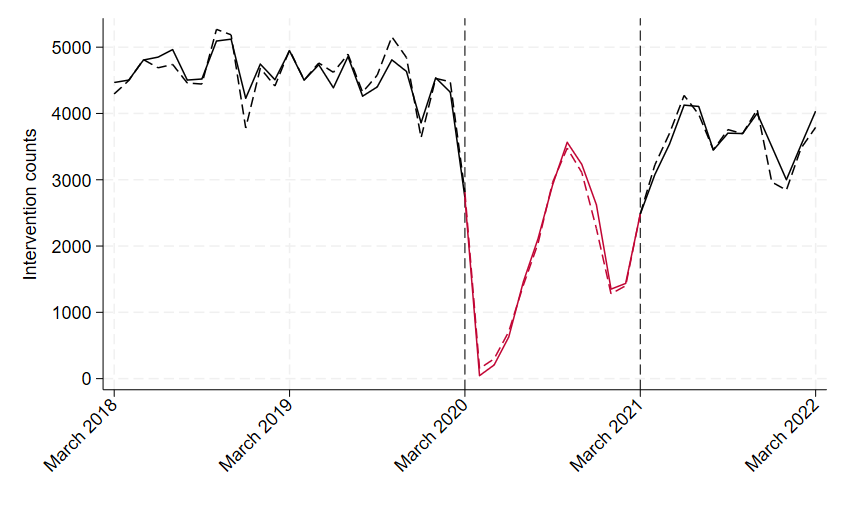
G. Hernia Repair (All Cat. 4 Control) H. Chronic Rhinosinusitis (ENT Cat. 4 Control)**

1. **Adenoids Removal (ENT Cat. 4 Control) J. Meniscal Tears. (MSK.Cat 4 Control)**

**
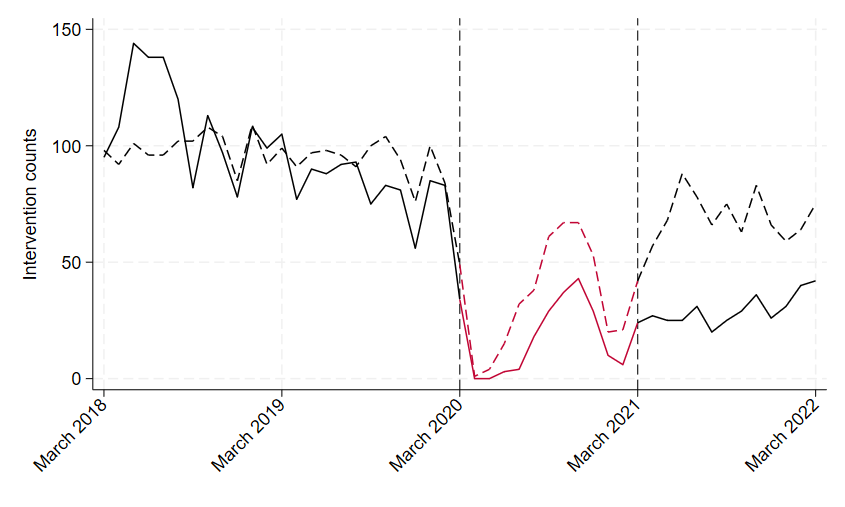

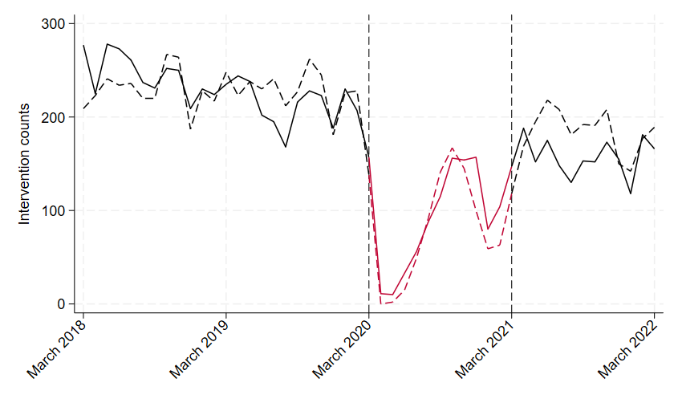
**

**
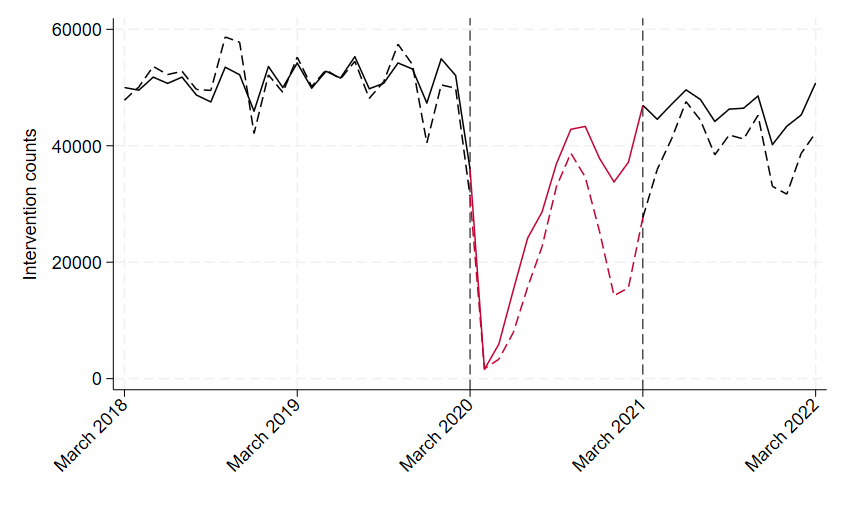

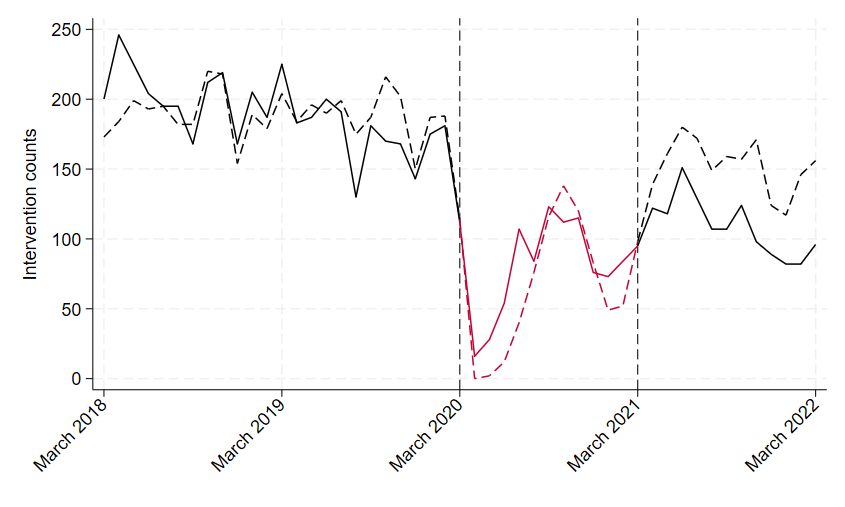
K. Lumbar Discectomy (MSK Cat. 4 Control) L. Upper GI Endoscopy (All Cat. 4 Control)**

**KEY**

EBI Procedures

Control Procedures

*Red sections represents excluded COVID timepoints*

| **EBI Procedure** | 19-20 | 20-21 | 21-22 | Pre/Post-COVID Diff  (19-20) –( 21-22) |
| --- | --- | --- | --- | --- |
| Exercise ECG | 84.0  (41.8 to 136.1) | 91.8  (41.4 to 158.1) | 60.6  (32.1 to 97.3) | -22.6  (-65.5 to 11.5) |
| Surgery for Chronic Rhinosinusitis | 1.7  (0.9 to 2.6) | 6.4  (1.8 to 13.9) | 2.6  (1.2 to 4.2) | 0.9  (-0.6 to 2.7) |
| Surgery for BPH | 7.2  (4.7 to 9.8) | 16.8  (10.6 to24.8) | 14.8  (16.8 to 107.3) | 7.5  (3.0 to 13.5) |
| Diagnostic Coronary Angiography | 5.6  (3.4 to 8.1) | 13.6  (5.1 to 29.5) | 8.1  (4.3 to 12.9) | 2. 4  (-0.6 to 6.1) |
| Cystoscopy | 65.9  (39.4 to 99.8) | 82.8  (42.1 to 136.8) | 71.8  (40.7 to 119.8) | 4.9  (-18.2 to 33.4) |
| Lumbar Discectomy | 30.5  (11.7 to 60.2) | 71.5  (21.7 to 141.3) | 54.8  (16.9 to 107.3) | 23.0  (0.1 to 58.4) |
| Adenoids Removal | 19.8  (8.9 to 33.0) | 32.0  (-6.6 to 102.8) | 37.3  (8.8 to 80.6) | 14.6  (-12.2 to 59.0) |
| Meniscal Tears | 23.6  (7.4 to 46.1) | 14.2  (6.1 to 24.0) | 30.4  (10.1 to 57.9) | 5.9  (-12.8 to 30.3) |
| RF Denervation | 94.7  (39.9 to 172.2) | 141.3  (31.5 to 336.3) | 85.2  (41.0 to 142.8) | -8.1  (-63.9 to 36.4) |
| Hernia Repair | 2.4  (0.9 to 4.7) | 9.8  (3.8 to 18.3) | 4.1  (1.7 to 7.3) | 1.7  (0.6 to 3.2) |
| Removal Kidney Stones | 3.5  (1.9 to 5.4) | 5.3  (2.8 to 8.3) | 3.4  (1.8 to 4.9) | -0.1  (-1.7 to 1.3) |
| Upper GI Endoscopy | 2.6  (1.7 to 3.7) | 3.2  (2.3 to 4.2) | 2.6  (1.7 to 3.4) | 0.0  (-0.7 to 0.7) |

Table S1 Variation in Procedure Rate across English ICBs: Systematic Component of Variation (SCV) Results

| **EBI Procedure** | Primary Analysis ^i^ | Model 1^ii^ | Model 2^iii^ | Model 3^iv^ |
| --- | --- | --- | --- | --- |
| Exercise ECG | -1,573**  (-1742, -1404) | -1605**  (-1768, -1442) | -1451**  (-1618, -1284) | -1573**  (-1742, -1404) |
| Surgery for Chronic Rhinosinusitis | -252**  (-298, -207) | -237**  (-284, -191) | -286**  (-324, -248) | -252**  (-298, -207) |
| Surgery for BPH | -102 **  (-171, -34) | -141**  (-203, -80) | -137  (-219, 56) | -103**  (-171, -34) |
| Diagnostic Coronary Angiography | -65  (-161, 31) | -86  (-185, 13) | -28  (-103, 48) | -65  (-160, 31) |
| Cystoscopy | -50  (-191., 92) | -103  (-243, 38) | -57  (-215, 101) | -50  (-191, 91) |
| Lumbar Discectomy | -43**  (-57, -29) | -42**  (-56, -27) | -45**  (-59, -31) | -43**  (-57, -28) |
| Adenoids Removal | -34 **  (-40, -28) | -34**  (-40, -28) | -31**  (-36, -26) | -34**  (-40, -28) |
| Meniscal Tears | -21*  (-42, 0) | -27.2**  (-48, -6) | -14  (-34, 6) | -21*  (-42, 0) |
| RF Denervation | 4.4  (-9, 18) | -1  (-14, 12) | 0  (-9, 9) | 4  (-9, 18) |
| Hernia Repair | 63  (-180, 306) | 44  (-200, 287) | -112  (-289, 64) | 63  (-180, 306) |
| Removal Kidney Stones | 231  (165, 297) | 261  (198, 324) | 208  (158, 258) | 231  (165, 297) |
| Upper GI Endoscopy | 3665  (1433, 5896) | 3710  (1475, 5945) | 4543  (3213, 5872) | 3665  (1433, 5896) |

Table S2 Time Periods Sensitivity Analyses

i One-year predictions from Table 1.

ii Model 1: COVID period starting 1^st^ February 2020

iii Model 2: COVID period extended to 31^st^ May 2020

^i^v Model 3: Interruption moved to November 2020

| **EBI Procedure** | Primary analysis (CITS) One Year Prediction | Lowest Priority Procedures Control Group One-Year Prediction |
| --- | --- | --- |
| Exercise ECG | -1,573.4**  (-1,742.4, -1,404.4) | -2,080**  (-2381, -1778) |
| Surgery for Chronic Rhinosinusitis | -252.3**  (-297.6, -206.9) | -67**  (-104, -30) |
| Surgery for BPH | 102.7**  (-170.9, -34.4) | 105.3**  (122.5, 178.1) |
| Diagnostic Coronary Angiography | -65.0  (-160.7, 30.6) | -372  (-555, 189) |
| Cystoscopy | -49.7  (-191.2, 91.8) | 308**  (233, 383) |
| Lumbar Discectomy | -42.7**  (-56.9, -28.5) | -70.3**  (-79, -62) |
| Adenoids Removal | -33.8**  (-39.9, -27.6) | -41.2**  (-53, -31) |
| Meniscal Tears | -21.0**  (-41.6, -0.3) | -45**  (-67, -22) |
| RF Denervation | 4.4  (-8.8, 17.6) | -7  (-17, 3) |
| Hernia Repair | 63.3  (-179.8, 306.3) | 46  (-47, 138) |
| Removal Kidney Stones | 230.7**  (164.8, 296.7) | 499**  (418, 579) |
| Upper GI Endoscopy | 3,664.7**  (1,433.0, 5,896.4) | 3,858  (-206, 7922) |

Table S3 Control Group Sensitivity Analyses
