# Supplementary material for: De-implementation of healthcare interventions post-COVID-19: evidence from the Evidence-Based Interventions (EBI) programme in England
Source: BMJ Open. 2026 Mar 30;16(3):e088256. doi: 10.1136/bmjopen-2024-088256 (PMC13052614; doi:10.1136/bmjopen-2024-088256)
Supplement: Supplementary file 3 [file bmjopen-2024-088256.reviewer_comments.pdf]

PEER REVIEW HISTORY

BMJ Open publishes all reviews undertaken for accepted manuscripts. Reviewers are asked to complete a checklist review form and are provided with free text boxes to elaborate on their assessment. These free text comments are reproduced below.

ARTICLE DETAILS

Title (Provisional)

De-adoption of healthcare interventions post-COVID: Evidence from the Evidence-Based Interventions (EBI) programme in England.

Authors

Glynn, Joel; Jones, Tim; Bell, Mike; Blazeby, Jane M; Burton, Christopher; Conefrey, Carmel; Donovan, Jenny; Farrar, Nicola; Morley, Josie; McNair, Angus GK; Owen-Smith, Amanda; Rule, Ellen; Thornton, Gail; Tucker, Victoria; Williams, Iestyn; Rooshenas, Leila; Hollingworth, William

---

VERSION 1 - REVIEW

**Reviewer**	**1**
**Name**	**Loke, Yoon**
**Affiliation**	**University of East Anglia, Norwich Medical School**
**Date**	**09-Jul-2024**
**COI**	**No competing interests**

Thank you for giving me the opportunity to review this interesting submission.

I must congratulate the authors for attempting to tackle the major challenges of evaluating trends in healthcare interventions over the tumultuous past 4 years.

There are therefore likely to be a large number of unmeasured confounding factors that undermine the study results, and would merit further discussion.

1) decreasing availability of GP appointments, and potentially different thresholds for referral to events where HES-APC codes arise. Differential rates of referral could arise if GPs chose to prioritise one procedure more than another. Is there another data source that gives numbers of GP consultations over the time periods of interest?

2) change in guidelines. I know that the authors have already mentioned that emergence of new trial data might have influenced the rates. However, it might be useful to check back 5 years (say, back to 2015) of NICE guidance or Expert Society guidance on the 12 procedures of interest to see if new advice had emerged.

3) emergence of different technology. The decrease in Exercise ECG may simply have been due to patients having different tests (cardiac MRI, radionuclide perfusion scans, CT coronary angiography) which are possibly more valid or reliable? It's not clear to me whether patients were shifted to a possibly better diagnostic modality, or whether there was a true reduction in patients being referred for this type of diagnostic testing. I guess it is not possible to easily answer this without a lot of additional work looking at HES to see if the decrease in exercise ECG was accompanied by parallel increases in demand for other coronary imaging.

**Reviewer**	**2**
**Name**	**Griffin, Daniel**
**Affiliation**	**Columbia University Irving Medical Center, Medicine**
**Date**	**25-Sep-2024**
**COI**	**none**

I appreciate the challenges of measuring such interventions surrounding the impact of the pandemic, but this remains critical. This paper is clear, well-written, and I appreciated the discussion regarding the absolute impacts of the interventions.

**Reviewer**	**3**
**Name**	**Kelson, Zoe**
**Affiliation**	**University of Exeter, Mathematics**
**Date**	**13-Feb-2025**
**COI**	**None**

This retrospective observational study explored the effectiveness of the Evidence-Based Interventions (EBI) programme which aims to reduce the number of inappropriate procedures or diagnostic tests (‘interventions’) in the English NHS.

Reviewer comments:

"utilising routine hospital data"

and

"Patients who had NHS-funded elective treatment in England from March 2018 to February 2022 inclusive"

and

"Data were extracted from Hospital Episode Statistics Admitted Patient Care (HES-APC) and Outpatient (HES-OP) datasets"

Can the authors please comment on whether there is any missing data?

"We generated unique control groups for each EBI Wave 2 intervention using the pool of elective interventions not considered for de-adoption, excluding cancer admissions. We used the Stata 'synth' command to generate control groups by selecting weighted averages of these interventions (captured as 3-character OPCS codes) [12]. This method created synthetic control groups that best resembled each EBI intervention in the pre-EBI-COVID period based primarily on the trend, but also taking account of monthly intervention counts, the proportion of women treated and average patient age"

and

"Using the control intervention trend from the model as a prediction of what would have happened without the EBI guidance"

The authors do well to perform a sensitivity analysis exploring different control group definitions.

"applied a controlled interrupted time series analysis to examine immediate and trend changes in expected EBI intervention counts following guidance publication" [Abstract]

and

"To explore whether trends in monthly intervention counts changed following the publication of the EBI guidance, we performed a controlled interrupted time series (CITS) analysis [15]. This analysis estimated whether there was a step (immediate) or trend (over time) change for each EBI intervention during the post-EBI-COVID period, compared to their control group"

and

"An interrupted time series (ITS) analysis was run using segmented linear regression on these differenced counts, with 1st March 2021 as the interruption point, to estimate step-change and trend-change coefficients for the CITS analyses. We included a winter month dummy variable to account for the usual seasonal drop in elective interventions."

and

"Indirectly age-sex standardised intervention rates per 100,000 residents were calculated for each year, taking account of population, age and sex differences between ICBs [16]... Two metrics were used to compare variation: the 90/10 ratio, and the systematic component of variation (SCV)[17]... We generated 95% confidence intervals around SCV statistics using the percentile method bootstrapping with 1,000 replications."

Appropriate modelling methods have been applied by the authors.

"Although the publication of EBI guidance was in November 2020, we assumed any step change would only become evident after elective interventions had largely recovered and levelled out at the start of the post-EBI-COVID period (i.e. 1st March 2021)"

The authors do well to perform a sensitivity on this period of lag between implementation and effectiveness.

"There are no universally agreed dates for when the effect of COVID-19 on elective interventions began or ended in England; therefore, we re-ran the CITS model with 1) an earlier start date (1st February 2020) for the COVID period; 2) a prolonged COVID period (1st March 2020 – 31st May 2021); and 3) an earlier interruption point (1st November 2020) assessing if the EBI guidance had any immediate impact on interventions. "

and

"We derived alternative control groups based on a clinical guide to surgical priorities during the COVID-19 pandemic which identified 'lowest priority' interventions during the pandemic [19]. For EBI interventions in specialities with a high volume of 'lowest priority' interventions (Urology; Musculoskeletal (MSK); Ear Nose and Throat (ENT)), we pooled all 'lowest priority' interventions in the relevant speciality as the control group. EBI interventions in other specialities were compared to a control group comprised of all 'lowest priority' interventions regardless of speciality (see online Supplement S2). We scaled the control group intervention rates to a similar level of monthly activity of each EBI intervention, using a multiplier generated by differencing average counts in the pre-EBICOVID period for both the EBI and control counts"

These sensitivity analyses help to demonstrate the robustness of the model findings.

"Our analyses assumed that pre-COVID-19 trends would have continued in a world without the pandemic. It is possible that the reductions in some EBI intervention counts by February 2022 were not achieved by EBI guidance alone." [strengths and limitations]

and

"Although our methods aimed to minimise the confounding effect of COVID-19, we cannot eliminate it. Our analyses assumed that pre-COVID-19 trends would have continued in a world without the pandemic. Although we believe this is a plausible assumption, it is possible that the reductions in some expected EBI intervention counts by February 2022 have been achieved by EBI guidance alone. The pandemic may well have been an important instigator for the de-adoption of procedures viewed as of lower value. However, our findings were largely consistent in sensitivity analyses when 'lowest priority' interventions not targeted by the EBI programme were used as control groups. This suggests that, at least for some procedures, EBI guidance may have played a role in reducing reuptake post-pandemic. Other concurrent factors, such as the publication of trial results, may also have affected the use of specific procedures. The synthetic control method allowed us to create control groups that closely matched EBI intervention trends pre-EBI-COVID for almost all interventions. However, the very high monthly counts for upper GI endoscopy limited the ability of this approach to select an appropriate control group. Our findings may be less reliable for this intervention, although we observed similar findings in sensitivity analysis using a 'lowest

priority' control group. There are no definitive start and end points for COVID-19 and its impact on health services. We used timepoints based on visual inspection of elective intervention counts over time. We also used sensitivity analysis to vary time points, finding little change in our results. Our analyses were limited to 12 of the 31 interventions targeted by the EBI programme. The other interventions were typically diagnostic tests and outpatient interventions that are not well recorded in routine data. We cannot generalise our findings to these interventions. "

A discussion on the study limitations has been provided

Can the authors please confirm if they adhered to any guidelines for the reporting of this study, and provide a copy of the associated checklist?

**Reviewer**	**4**
**Name**	**Cohen, Rubin**
**Affiliation**	**Syracuse VA Medical Center, Medicine**
**Date**	**24-Jun-2025**
**COI**	**none**

The manuscript discusses an important issue in healthcare, namely the use of limited resources and whether the presence of guidelines impacts inappropriate care.

A few points:

I found the abstract rather difficult to read. I wonder if the authors can revise it.

The statistics are beyond this reviewer's expertise. I am unable to comment.

I agree that COVID-19 presented a unique opportunity. The majority of elective procedures were cancelled during the pandemic. Some procedures, while increasing post pandemic, did not reach the numbers noted pre-pandemic. This may be due to guidelines, or maybe due slower than expected return to "normal". In some instittions, many healthcare workers retired, especially nurses. It took some time to hire an dtrain new staff. Thus, it took time for the number of procedures to return to pre-pandemic levels.

As the authors discuss, EBI may have a limited effect on established practice patterns, and ".....many questions remain about the mechanisms by which de-adoption programmes work and why they work in some circumstances and not others". Now that the authors established the changes their study, with its limitations, it would be interesting to explore these questions further in subsequent research.

**Reviewer**	**5**
**Name**	**Ranapurwala, Shabbar**

Affiliation **University of North Carolina at Chapel Hill, Department of Epidemiology**

Date **21-Jul-2025**

COI **None**

Thanks for the opportunity to review this work, which seems quite important, and the authors have put in a lot of time and effort here. However, I have a few concerns regarding the methodology of the paper. In my general view, the authors have overly simplified some of the analysis and overly complicated others. Here are some pointers that can provide guidance for modification if the authors choose to do so:

1. Why were the 14 partially measurable ones not examined in this study - not doing this work leaves out important understanding regarding the effectiveness of the EBI program.

2. Using counts rather than rates is problematic because,

a. The counts assume that the patients suffering from a specific condition are constant over time, but COVID infection may have increased or decreased propensity of some health conditions and it will be changing over time as more and more people are infected with COVID.

b. Even without the COVID-19 pandemic, assuming that the propensity of various health conditions for which these intervention are provided remains constant is difficult to imagine, and the authors have provided no data to support that assumption.

3. Authors say, "Our analyses assumed that pre-COVID-19 trends would have continued in a world without the pandemic." But they don't have to make this assumption - all they need to do is to conduct a full controlled interrupted time series analysis with two interventions, rather than using count differences and single series ITS. This limitation is because of how the analyses were conducted, even though the authors have the data and better methods at their disposal to not make such assumptions.

a. Currently by using single series ITS, based on figure 2, the authors are comparing pre-EBI-COVID period to post-EBI-COVID period, while ignoring the changes that occurred during COVID, that could have been different between two groups as well (and that can be seen in figures 3A-F).

b. The current analyses assumes that the effect of COVID on the intervention and the synthetic control group was the same. The reason we use controls in CITS analysis is simply to avoid such issues, where the different impact of COVID among the groups can be accounted for. See this study: https://pubmed.ncbi.nlm.nih.gov/40484630/ , for a recent example of two interruptions used in relation to COVID.

4. Some of the 12 "interventions" are actually health conditions instead of interventions. E.g., "Chronic Rhinosinusitis" or "Meniscal tears"

5. Authors offer very limited interpretation of the data in the discussion. Does not go into detail as to why the intervention really worked in 1 of 12 selected interventions only, and what does that mean for the EBI program? why the particular intervention was impacted and not others?

6. The interruption used in the study was March 2021, that the authors assume as the end of the pandemic, but this is problematic, because the most virulent form of COVID-19, the Omicron variant, was first identified in November 2021, hence even the end date of the entire study (Feb 2022) was also during the pandemic. The simplest analyses would have been to use the interventions as they appeared, e.g., the EBI wave 2 announcement in November 2020. Those results, combined with CITS should become the primary analysis, and others can be supplemental sensitivity analyses.

VERSION 1 - AUTHOR RESPONSE

Reviewer: 1
Prof. Yoon Loke, University of East Anglia
Comments to the Author:
Thank you for giving me the opportunity to review this interesting submission.

I must congratulate the authors for attempting to tackle the major challenges of evaluating trends in healthcare interventions over the tumultuous past 4 years.
There are therefore likely to be a large number of unmeasured confounding factors that undermine the study results, and would merit further discussion.

A number of approaches were taken to minimise confounding and identify any impact of the EBI programme. These are detailed in our Methods and Discussion sections of the paper.

The main method was to include control groups identified using two methods.

1.) Synthetic controls
2.) 'Lowest Priority' procedure controls by specialty.

Controlling for similar elective interventions not targeted by the EBI programme reduces the impact of unmeasured confounding given that some external shocks (e.g. COVID-related hospital ward closures or re-purposing) affected all elective hospital care. Any differences observed between intervention and control groups may, therefore, plausibly be due to the EBI programme. We discuss these methods and other potential confounders (e.g. publication of new evidence) in the limitations section of the discussion.

1) decreasing availability of GP appointments, and potentially different thresholds for referral to events where HES-APC codes arise. Differential rates of referral could arise if GPs chose to prioritise one procedure more than another. Is there another data source that gives numbers of GP consultations over the time periods of interest?

The number of GP appointments fluctuated over the study period [1], although there is no substantial increase or decrease (except during the COVID period), which would plausibly have a differential effect on referral rates for EBI and control procedures post-COVID.

2) change in guidelines. I know that the authors have already mentioned that emergence of new trial data might have influenced the rates. However, it might be useful to check back 5 years (say, back to 2015) of NICE guidance or Expert Society guidance on the 12 procedures of interest to see if new advice had emerged.

The EBI programme published guidelines to support its recommendations that included the most recent evidence, NICE, and other organisational guidelines [2]. The EBI guidelines are referred to in the paper (Line 144). We do not believe that an additional review of NICE and Specialist society guidance would add sufficient value to the paper. It is possible that other guidelines were published post-EBI and we have acknowledged this as a potential limitation (Page 13 Lines 325- 326).

3) emergence of different technology. The decrease in Exercise ECG may simply have been due to patients having different tests (cardiac MRI, radionuclide perfusion scans, CT coronary angiography) which are possibly more valid or reliable? It's not clear to me whether patients were shifted to a possibly better diagnostic modality, or whether there was a true reduction in patients being referred for this type of diagnostic testing. I guess it is not possible to easily answer this without a lot of additional work looking at HES to see if the decrease in exercise ECG was accompanied by parallel increases in demand for other coronary imaging.

The goal of the EBI programme was/is not only to reduce overall activity, but move people and resources to more appropriate care. Going into more detail for each procedure would be fascinating, however beyond the scope and practical space of a single paper. The aim of this work was to capture high level evidence of whether reduction in the procedures and geographic variation were achieved following the publication of EBI guidance.

Reviewer: 2
Dr. Daniel Griffin, Columbia University Irving Medical Center
Comments to the Author:
I appreciate the challenges of measuring such interventions surrounding the impact of the pandemic, but this remains critical. This paper is clear, well-written, and I appreciated the discussion regarding the absolute impacts of the interventions.

Thanks for this encouraging comment.

Reviewer: 3
Prof. Zoe Kelson, University of Exeter
Comments to the Author:
This retrospective observational study explored the effectiveness of the Evidence-Based Interventions (EBI) programme which aims to reduce the number of inappropriate procedures or diagnostic tests ('interventions') in the English NHS.

Reviewer comments:

Can the authors please comment on whether there is any missing data?

We have added details in the methods section (Lines 156 -160). One of the key strengths of the work is the use of HES data that has very limited missing data given its use as the basis for reimbursement in the English NHS.

The authors do well to perform a sensitivity analysis exploring different control group definitions.

No response required

Appropriate modelling methods have been applied by the authors.

No response required

The authors do well to perform a sensitivity on this period of lag between implementation and effectiveness.

No response required

These sensitivity analyses help to demonstrate the robustness of the model findings.

No response required

A discussion on the study limitations has been provided

No response required

Can the authors please confirm if they adhered to any guidelines for the reporting of this study, and provide a copy of the associated checklist?

Apologies, a 'Record Checklist' was completed alongside the writing of this paper, but was missed off the original submission. This has now been included.

Reviewer: 4
Dr. Rubin Cohen, Syracuse VA Medical Center, SUNY Upstate Medical University
Comments to the Author:
The manuscript discusses an important issue in healthcare, namely the use of limited resources and whether the presence of guidelines impacts inappropriate care.

A few points:
I found the abstract rather difficult to read. I wonder if the authors can revise it.

The abstract has been revised to make it clearer.

The statistics are beyond this reviewer's expertise. I am unable to comment.
I agree that COVID-19 presented a unique opportunity. The majority of elective procedures were cancelled during the pandemic. Some procedures, while increasing post pandemic, did not reach the numbers noted pre-pandemic. This may be due to guidelines, or maybe due slower than expected return to "normal". In some instittions, many healthcare workers retired, especially nurses. It took some time to hire an dtrain new staff. Thus, it took time for the number of procedures to return to pre-pandemic levels.
As the authors discuss, EBI may have a limited effect on established practice patterns, and "…..many questions remain about the mechanisms by which de-adoption programmes work and why they work in some circumstances and not others". Now that the authors established the changes their study, with its limitations, it would be interesting to explore these questions further in subsequent research.

The study presented in this paper is one part of a large mixed-methods evaluation of the EBI programme in which we are exploring the delivery, impact and acceptability of the initiative. Our wider project aims to tease out some of the mechanisms that explain the EBI programme's impact and de-implementation more generally. Some of this work has been published [3, 4], with other papers forthcoming. We discuss this in the future research section of the discussion.

Reviewer: 5
Dr. Shabbar Ranapurwala, University of North Carolina at Chapel Hill
Comments to the Author:
Thanks for the opportunity to review this work, which seems quite important, and the authors have put in a lot of time and effort here. However, I have a few concerns regarding the methodology of the paper. In my general view, the authors have overly simplified some of the analysis and overly complicated others. Here are some pointers that can provide guidance for modification if the authors choose to do so:

1. Why were the 14 partially measurable ones not examined in this study - not doing this work leaves out important understanding regarding the effectiveness of the EBI program.

We understand this concern and have added more detail in the Methods and Discussion sections (Page 13 lines 343 - 352). The activity rates for these 14 procedures cannot be accurately measured using routine healthcare data. Undertaking an analysis of these 14 procedures would have produced inconclusive and likely misleading results. We have extended the limitations section of the discussion to acknowledge this.

Nevertheless, we believe that an evaluation of the 12 "measurable" interventions provides important understanding of the effectiveness of the EBI programme. When considered alongside findings from our published analysis of 17 EBI Wave One procedures [5], and qualitative work [3, 4], we believe it provides useful insights into the challenges faced by the EBI programme that will inform future de-implementation initiatives.

2. Using counts rather than rates is problematic because,

a. The counts assume that the patients suffering from a specific condition are constant over time, but COVID infection may have increased or decreased propensity of some health conditions and it will be changing over time as more and more people are infected with COVID.

Please see answer to 2b below.

b. Even without the COVID-19 pandemic, assuming that the propensity of various health conditions for which these intervention are provided remains constant is difficult to imagine, and the authors have provided no data to support that assumption.

While it is possible that the prevalence of the 12 conditions changed substantially over the study period, we used counts rather than rates because:

I. There are no accurate data source capturing time trends for the prevalence of these conditions over this time-period. Using inaccurate data would introduce bias into our analyses.

II. The pandemic affected fitness, nutrition, mental health, etc. and this in turn may have impacted the prevalence of conditions related to the EBI procedures. However, we have no reason to believe that it did so more than for other conditions related to the control procedures.
III. Having a control group for each procedure, we were able to account for changes in referral and capacity issues that resulted from the pandemic more generally.

We have added a paragraph to our limitations to discuss this point (Page 13 Lines 329-336)

3. Authors say, "Our analyses assumed that pre-COVID-19 trends would have continued in a world without the pandemic." But they don't have to make this assumption - all they need to do is to conduct a full controlled interrupted time series analysis with two interventions, rather than using count differences and single series ITS. This limitation is because of how the analyses were conducted, even though the authors have the data and better methods at their disposal to not make such assumptions.

a. Currently by using single series ITS, based on figure 2, the authors are comparing pre-EBI-COVID period to post-EBI-COVID period, while ignoring the changes that occurred during COVID, that could have been different between two groups as well (and that can be seen in figures 3A-F).

Figure 2 is an illustrative example of how we handled the COVID-19 period and does not represent our analysis which was a full CITS analysis using synthetic controls. We choose not to model the period from February 2020 to February 2021 as the primary aim of our paper was to identify any longer-term impact of the EBI programme beyond the COVID period rather than within it. Using the full study period (including COVID) would have significantly complicated the analysis as it would require to additional 'interruptions' (e.g. the start and end of COVID restrictions) in close succession to be modelled. Given the primary aim of our paper, we opted for a simpler CITS model.

b. The current analyses assumes that the effect of COVID on the intervention and the synthetic control group was the same. The reason we use controls in CITS analysis is simply to avoid such issues, where the different impact of COVID among the groups can be accounted for. See this study: https://pubmed.ncbi.nlm.nih.gov/40484630/ , for a recent example of two interruptions used in relation to COVID.

We read the paper with interest. We believe that the difference in methodological approach is largely due to a difference in aims. As stated above, we were primarily interested in the longer-term impact of the EBI programme beyond the COVID period rather than within it. The EBI guidance was designed to have a long-term impact on use of procedures. In contrast, the paper cited aims to distinguish the immediate (Mar-May 2020) and medium-term (during the remainder of 2020) impact of stay at home (SAH) orders on suicide rates during the COVID crisis. SAH was a short-term temporary measure introduced to reduce the spread of infection.

4. Some of the 12 "interventions" are actually health conditions instead of interventions. E.g., "Chronic Rhinosinusitis" or "Meniscal tears"

Thank you for spotting these. All 12 interventions are surgical procedures and we have edited the shorthand labels used in our text to make this clear.

5. Authors offer very limited interpretation of the data in the discussion. Does not go into detail as to why the intervention really worked in 1 of 12 selected interventions only, and what does that mean for the EBI program? why the particular intervention was impacted and not others?

Within the scope of this paper, we do not have space to speculate on the factors underlying the trends observed for all 12 procedures. In response to this comment, we have added more detail about Exercise ECG where we observed the largest difference in predicted procedures at 1 year. We note that this was the only analysed procedure that was given a “do not do” recommendation rather than restrictive criteria for appropriate access. (Page 14 Lines 382 - 385). We cite other work from the wider project that explores reasons why the EBI guidance struggled to have a more universal impact.

6. The interruption used in the study was March 2021, that the authors assume as the end of the pandemic, but this is problematic, because the most virulent form of COVID-19, the Omicron variant, was first identified in November 2021, hence even the end date of the entire study (Feb 2022) was also during the pandemic. The simplest analyses would have been to use the interventions as they appeared, e.g., the EBI wave 2 announcement in November 2020. Those results, combined with CITS should become the primary analysis, and others can be supplemental sensitivity analyses.

As demonstrated in Figure 1b the Omicron variant, which occurred five months after all UK adults were able to get a first vaccine dose, had little impact on elective hospital procedure counts. We don't assume the pandemic ended in March 2021, but that the impact of COVID on elective surgical capacity in England had largely resolved by March 2021 such that any longer-term impact of EBI guidance (compared to the control group in the CITS) could be observed. We disagree that modelling an EBI interruption in Nov 20 would have resulted in a simpler analysis or clearer interpretation. In sensitivity analyses (Table S2, model 3), we explored moving the interruption point to November 2020 and our primary findings are unchanged.

References

1. Government If. Performance Tracker 2022/23: Spring update - General practice. Institute for Government2023.
2. Academy of Medical Royal Colleges: About Us https://ebi.aomrc.org.uk/about-us/ (2024). Accessed 15th January 2024.
3. Conefrey C, Farrar N, Coyle M, Bell M, Blazeby J, Burton C, et al. Delivering a national de-adoption programme: a documentary analysis of local commissioning policy compliance with England's Evidence-based Interventions programme (EBI). BMC Health Serv Res. 2025;25(1):995. doi: 10.1186/s12913-025-13012-0.
4. Farrar N, Conefrey C, Bell M, Blazeby J, Burton C, Donovan J, et al. Relevance and flexibility are key: exploring healthcare managers' views and experiences of a de-adoption programme in the English National Health Service. BMC Health Serv Res. 2025;25(1):590. doi: 10.1186/s12913-025-12700-1.
5. Glynn J, Jones T, Bell M, Blazeby J, Burton C, Conefrey C, et al. Did the evidence-based intervention (EBI) programme reduce inappropriate procedures, lessen unwarranted variation or lead to spill-over effects in the National Health Service? PLoS One. 2023;18(9):e0290996. doi: 10.1371/journal.pone.0290996.

VERSION 2 - REVIEW

**Reviewer**	**3**
**Name**	**Kelson, Zoe**
**Affiliation**	**University of Exeter, Mathematics**
**Date**	**12-Nov-2025**
**COI**	

Many thanks to the authors for their reponses and revisions.
